# Transperitoneal laparoscopic umbilical resection of urachal remnants: a feasible surgical method

**DOI:** 10.1186/s12894-023-01229-2

**Published:** 2023-04-04

**Authors:** Hideo Yuki, Naoya Ohkubo, Ryo Kurashina, Kazumasa Sakamoto, Issei Suzuki, Kohei Takei, Hironori Betsunoh, Akinori Nukui, Masahiro Yashi, Takao Kamai

**Affiliations:** 1 Department of Urology, Dokkyo Medical University Nikko Medical Center, 145-1 Moritomo, Nikko, Tochigi 321-1298 Japan; 2 Department of Urology, Dokkyo Medical University, 880 Kitakobayashi, Mibu-Machi, Shimotsuga-gun, Tochigi 321-0293 Japan; 3 Department of Urology, Tochigi Cancer Center, 4-9-13 Yonan, Utsunomiya, Tochigi 320-0834 Japan

**Keywords:** Laparoscopic resection, Surgical treatment, Urachal remnant, Umbilicus, Urachal sinus

## Abstract

**Background:**

To date, there is no standard established laparoscopic surgical method for managing urachal remnants because of their rarity, and several questions remain unanswered. Are there any problems for considering the operative indications about patients’ factors for example, body mass index and so on? This study aimed to determine the feasible surgical method for managing urachal remnants and presents the operative outcomes of our cases in relation to the findings from the existing literature.

**Methods:**

We analyzed the data of 16 patients (7 women and 9 men; age range, 19–48 years) who underwent surgery for urachal remnants between January 2013 and March 2019 at our institution.

**Results:**

In our cases, all urachal remnants were urachal sinuses, and the primary complaints were umbilical pain and pus discharge. Most of these symptoms were controlled using umbilical drainage and oral antibiotic intake; however, incisional drainage was required in two cases. In all cases, we performed a laparoscopic resection of the urachal remnants; one patient underwent an open conversion due to a very thick abdominal wall. Therefore, “peri-umbilical distanse” was proposed as an index to verify the periumbilical abdominal wall thickness. This index may clear the difficulties of the laparoscopic resection of the urachal remnunts. A postoperative complication—local infection that was treated using re-suturing—was observed in one patient. No adverse events occurred in the other cases. Our method was appropriate because it allowed for complete urachal resection with good cosmetic results, i.e., a small and natural scar appearance. Additionally, if bladder injury occurred, bladder re-suturing was easily possible because of the laparoscopic port’s position.

**Conclusions:**

We present an feasible method for laparoscopic urachal resection. This method may be recommended for young patients with an peri-umbilical distanse of < 2 cm.

**Supplementary Information:**

The online version contains supplementary material available at 10.1186/s12894-023-01229-2.

## Background

The urachal remnant is a rare congenital anomaly [1]. The urachus is a duct, present before birth, that serves as a communication between the umbilicus and bladder. This duct usually disappears after birth, leaving behind fibrous tissue. Full-length urachal remnants, such as patent urachus, are observed in rare cases. The urachal sinus and vesicourachal diverticulum are observed at the sides of the umbilicus and bladder, respectively, and the urachal cyst is observed at the midline. Conservative treatment of infected urachi with antibiotics as the only form of therapy risks re-infection [2]. Thus, complete resection of the residual pouch (duct) is the most appropriate form of treatment.

While several surgical procedures (such as open, laparoscopic, and retroperitoneoscopic procedures) are available for urachal remnants [3–6], the best method for endoscopic surgery remains undetermined. For example, the best port position and appropriate approach (peritoneal or retroperitoneal) remain unclear. This study aimed to determine the operative indications of the patients’ factors, effective surgical approach to repairing urachal remnants and present the operative outcomes of our cases in relation to the findings from the existing literature.

## Methods

### Patient characteristics

This study included 16 patients (seven women and nine men) with urachal remnants, who were referred to our hospital, and subsequently underwent surgical management between January 2013 and March 2019. All patients had urachal sinus infections. The patients were aged between 19 and 48 years (Table 1).

**Table 1:** Age, sex, and BMI of each patient enrolled in the study

No	Age	Sex	BMI
1	30	F	16.52
2	30	M	19.79
3	33	F	20.03
4	25	M	23.21
5	39	M	16.78
6	25	M	36.16
7	27	F	21.56
8	46	M	20.25
9	34	F	23.15
10	22	F	17.69
11	24	M	21.03
12	19	F	22.17
13	23	M	25.97
14	48	M	19.12
15	32	M	19.61
16	24	F	18.44

*M*, male; *F*, female; *BMI*, body mass index

### Surgical technique

**Fig. 1:**
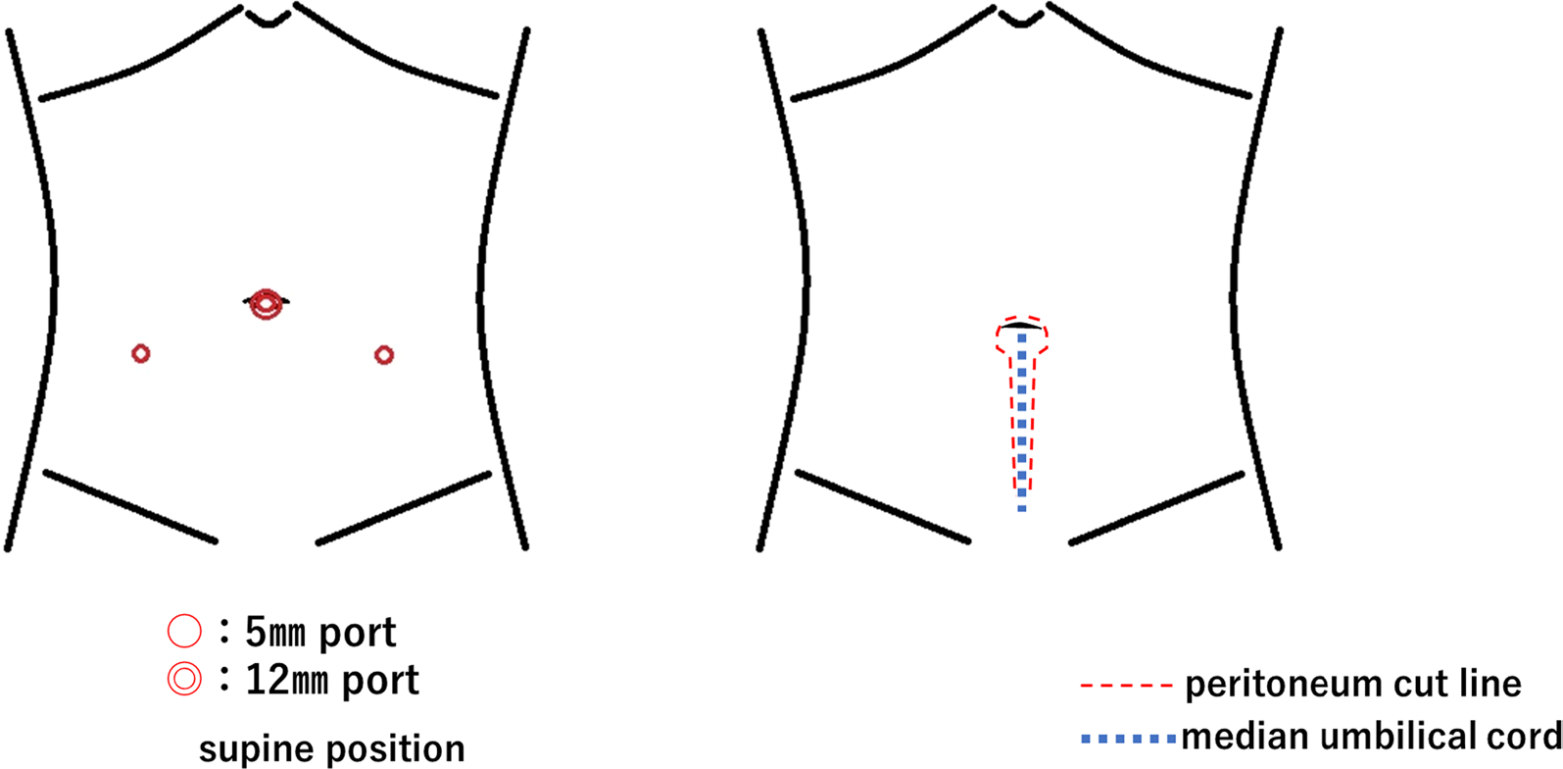
The port positions and cut line of the peritoneum

The surgical approach was transperitoneal. We began the umbilical resection from the inner part of the umbilicus. The caudal part of the umbilicus was not resected because of the presence of the urachus. In our method, we resected both the umbilicus and the urachus. The umbilicus and urachus were lowered into the peritoneal cavity. The resection site was used as a port for a 12-mm camera (Fig. 1). Before setting the port, we detached the caudal part of the hollow umbilicus and urachus from the abdominal wall. This method made endoscopic visualization easier because the endoscope was away from the target (urachus). After setting the camera port, a bilateral 5-mm port was set beside the rectus abdominis muscle. The urachus was resected from the abdominal wall using an endoscopic technique (Fig. 1). The bilateral medial umbilical folds were cut, and the caudal end of the urachus was cut near the bladder dome. The resected bladder dome was repaired by suturing. The resected peritoneum was not repaired, and the resected urachus was excised in a plastic bag. We cautiously closed the incision using an absorbable suture, considering the aesthetics of the scar. The scar appeared as a natural umbilicus after surgery owing to the hollowing of the umbilicus into the peritoneal cavity.

### Statistical analysis

The Spearman’s rank and Mann–Whitney U tests were used the correlation and comparative analyses, respectively. All statistical analyses were performed with EZR (Saitama Medical Center, Jichi Medical University, Saitama, Japan), which is a graphical user interface for R (The R Foundation for Statistical Computing, Vienna, Austria).

## Results

**Fig. 2:**
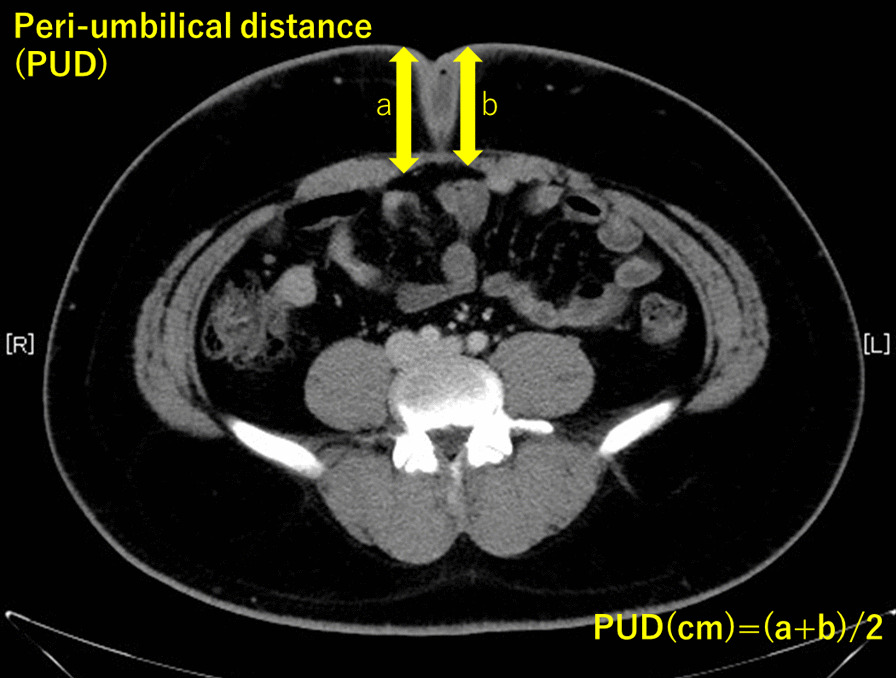
Peri-umbilical distance shows the thickness of the abdominal wall

All patients' symptoms were pain and discharge, and all patients' urachus type was urachal sinus. We performed the surgeries after controlling the infections. The median follow-up period was 126 days (range, 84–251 days), as shown in Table 2. Complications, such as umbilical infection due to a urachal sinus, were observed in all cases of the remaining duct. One patient with obesity (body mass index, 36.2 kg/m^2^) underwent a conversion to open surgery because of a very thick abdominal wall (5.9 cm, Fig. 2). In addition, the peritoneal cavity was very small; after setting the camera port, the cavity could not be inflated with carbon dioxide gas.

**Table 2:** Patient presurgical, surgical, and postsurgical characteristics

No	First visit ~ ope (days)	Ope time (min)	Pneumo-peritoneum time (min)	Operator	Ope ~ discharge time (days)	Bladder incision + suturing	Clavien Dindo	PUD (cm)
1	103	166	79	A	6	y	II	2.43
2	165	212	120	A	7	y	–	2.23
3	105	174	120	A	7	y	–	1.84
4	196	107	52	B	21	n	IIIa	3.71
5	206	88	36	A	6	n	–	1.46
6	84	247	55	A	9	y	IIIa	5.89
7	105	111	44	A	4	n	–	2.77
8	158	83	27	A	7	n	–	1.42
9	186	73	20	A	3	n	II	2.98
10	251	79	27	A	9	n	–	1.46
11	113	83	29	A	4	n	–	1.57
12	130	97	38	A	4	n	–	3.22
13	126	140	64	A	6	n	–	2.99
14	85	81	23	A	6	n	–	2.25
15	117	68	19	A	3	n	–	2.61
16	85	86	30	C	3	n	–	1.51

Perioperative adverse events were observed in one patient who developed a surgical site infection on postoperative day 3. After controlling the infection, the site was re-sutured. This infection was attributed to umbilical perforation during the surgery. See supplementary information for other data (Additional file 1).

## Discussion

The urachus is found between the umbilicus and bladder during the prenatal period. In normal cases, the urachus closes and becomes the median umbilical ligament before birth. The urachal remnant is the remaining urachus after birth [7] and is associated with umbilical infection. Local treatment such as drainage or the use of antibiotics is effective; nonetheless, 30% of the patients who receive such treatment experience re-infection [8]. Therefore, the curative treatment is resection of the urachal remnant.

Recently, endoscopic resection has been commonly used; however, a standard method for laparoscopic urachal resection is lacking. Both the peritoneal and retroperitoneal methods have been suggested [3–6].

It is easy to identify the urachus using the peritoneal approach because the urachus adheres to the peritoneum. During surgery, resecting the urachus along with the peritoneum is easy, provided that the peritoneal cavity is large enough for the endoscopic technique. However, this approach is associated with a risk of intestinal injury and postoperative intestinal adhesions. The retroperitoneal approach is the most appropriate method for reducing the risk of intestinal injury and adhesions. However, the cavity is often too small to set the port. As described above, the urachus adheres to the peritoneum, and separating them is difficult. Locating the urachus near the umbilicus is particularly difficult because the urachus is very thin.

The optimal position of the port remains undetermined. In our method, there were three ports: the umbilical camera port and left and right bilateral abdominal ports. This position was reasonable because of the endoscopic triangle. Considering the central camera, the central target organ and bilateral forceps were appropriate for endoscopy and provided the best ergonomic positioning. Urachal resection was easy, especially around the bladder. However, the disadvantage of this position was the initial part of the endoscopy. After setting the camera and bilateral ports, the camera and the urachus were close to each other. In endoscopic surgery, it is difficult to resect a close target because of the reduced visual field; therefore, hollowing of the umbilicus before endoscopy is crucial.

**Fig. 3:**
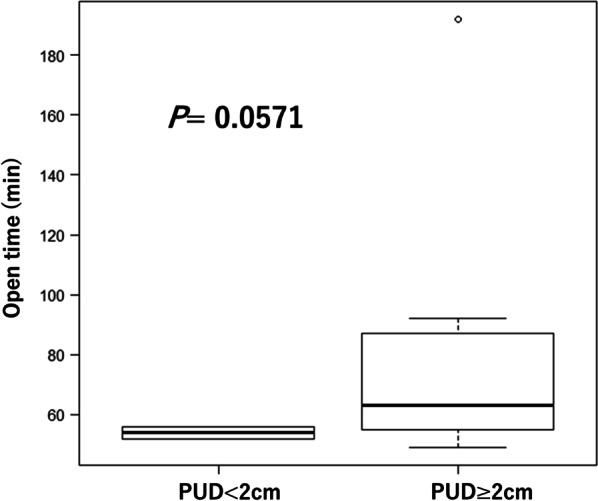
Difference of the approach time from the beginning of operation to start of laparoscopic procedure

**Fig. 4:**
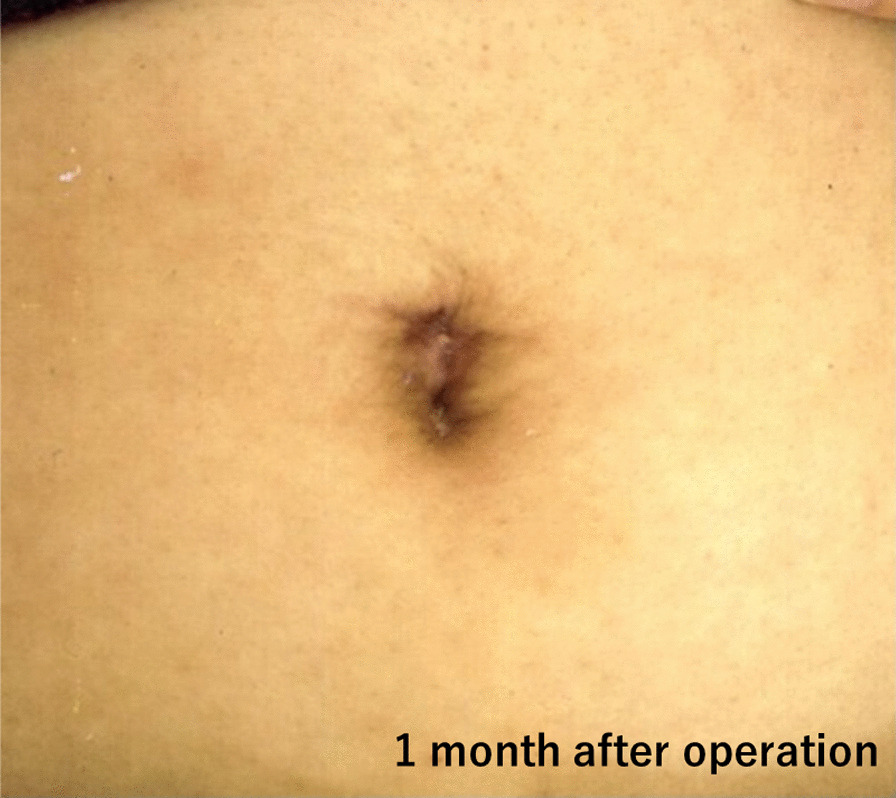
The final umbilical appearance after the surgery

**Fig. 5:**
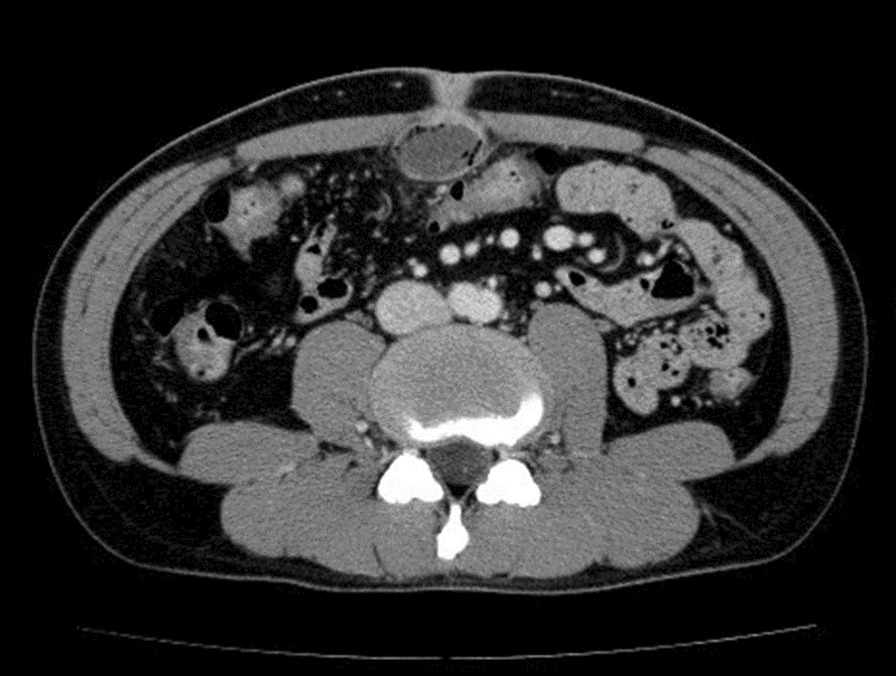
A large abscess was forming subcutaneously

**Fig. 6:**
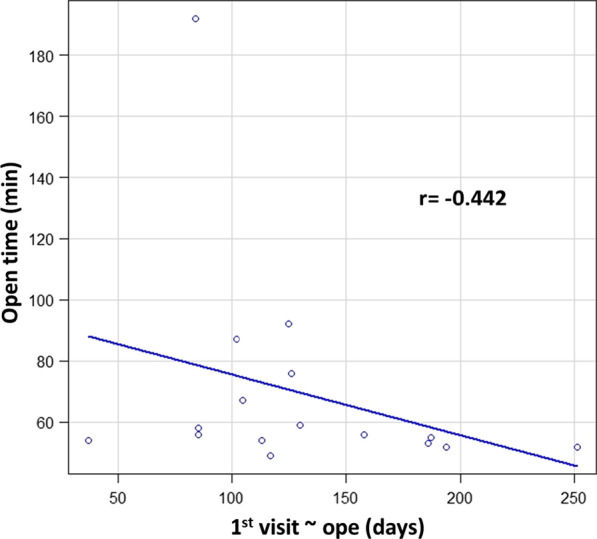
The relevance between the open time and period from first visit to surgery. Relation was observed (r = − 0.442)

Three significant points need to be considered for laparoscopic urachal resection. The first is the thickness of the abdominal wall (peri-umbilical distanse: PUD). In our case, a thick abdominal wall was one of the difficulties that we encountered; the time for the initial part of the operative correlated with wall thickness (data not shown). Patients with an PUD > 2 cm were more likely to have longer operative times (Fig. 3). In patients with an PUD > 2 cm, setting the umbilical camera port was difficult; thus, we considered changing the camera port to the lateral abdominal port [9]. The second point is cosmetics. Most of the patients in our study were young. The appearance of the surgical site is critical, especially for young women. Although the urachal remnant is not malignant, caution should be exercised when performing surgery to ensure patient satisfaction and improvement of the quality of life after the operation. Our umbilical repair method is easy to perform, and the umbilicus retains a natural appearance; the lateral ports used are 5 mm in size, which is appropriate for achieving good cosmetic results (Fig. 4). Considering cosmetic aspects, the laparoendoscopic single-port surgery may be another suitable option [6, 10, 11]. The third point is infection control before surgery. The severity of umbilical infection was not related to the success of the surgery. In Case 8 (Table 1, Fig. 5), the infection was very severe, requiring drainage and debridement. Five months after this treatment, successful urachus resection was performed within the median operative time, without any adverse events (Table 2). Infection control for at least 4 months before the operation is recommended (Fig. 6).

This study had a few limitations. First, it included a small number of cases, resulting in an insufficient number of treatment options. Second, the patients’ quality of life was not evaluated in this study. Future studies should enroll more patients and evaluate patient satisfaction after the procedure. The laparoscopic approach before open surgery could be performed safely for the majority of surgical operations nowadays. Laparoscopic minimally invasive surgery should be preferred over open surgery due to its advantages [12].

In conclusion, we present the laparoscopic as the feasible method for urachal resection. This method may be recommended for young patients with an PUD of < 2 cm. In addition, sufficient time (> 4 months) is required for infection control before the operation.

## Supplementary Information

**Additional file 1.** Additional detailed data for all cases are presented.

## Data Availability

The data that support the findings of this study are available from the corresponding author [HY] upon reasonable request.

## Abbreviation

BMI: Body mass index

## References

[1] Siow SL, Mahendran HA, Hardin M (2015). Laparoscopic management of symptomatic urachal remnants in adulthood. Asian J Surg.

[2] Araki M, Saika T, Araki D, Kobayashi Y, Uehara S, Watanabe T (2012). Laparoscopic management of complicated urachal remnants in adults. World J Urol.

[3] Yohannes P, Bruno T, Pathan M, Baltaro R (2003). Laparoscopic radical excision of urachal sinus. J Endourol.

[4] Khurana S, Borzi PA (2002). Laparoscopic management of complicated urachal disease in children. J Urol.

[5] Patrzyk M, Glitsch A, Schreiber A, von Bernstorff W, Heidecke CD (2010). Single-incision laparoscopic surgery as an option for the laparoscopic resection of an urachal fistula: first description of the surgical technique. Surg Endosc.

[6] Ishii K, Sakamoto W, Yamamoto T, Nishihara C (2019). Initial experience with laparoscopic single-site retrograde urachal resection for urachal remnant using a retroperitoneal approach for pediatric cases. Int J Urol.

[7] Blichert-Toft M, Nielsen OV (1971). Diseases of the urachus simulating intra-abdominal disorders. Am J Surg.

[8] Blichert-Toft M, Nielsen OV (1971). Congenital patient urachus and acquired variants. Diagnosis and treatment. Review of the literature and report of five cases. Acta Chir Scand.

[9] Maemoto R, Matsuo S, Sugimoto S, Tokuka A (2019). Umbilical resection during laparoscopic surgery for urachal remnants. Asian J Endosc Surg.

[10] Narita T, Kunimitsu A, Takahashi J, Asami K, Takahashi K, Sato T (2016). Three cases of umbilical urachal sinus treated with single-incision laparoscopic surgery Japanese. J Endourol.

[11] Hoshi A, Chihara I, Shiga M, Nitta S, Nagumo Y, Sakka S (2022). Laparoendoscopic single-site surgery for urachal remnant with extraperitoneal approach through a suprapubic port. Asian J Endosc Surg..

[12] Mulita F, Papadopoulos G, Tsochatzis S, Kehagias I (2020). Laparoscopic removal of an ingested fish bone from the head of the pancreas: case report and review of literature. Pan Afr Med J.

